# Renal Metabolic Rate of Oxygen in Response to Hypoxia Challenges by Means of Quantitative MRI in Humans

**DOI:** 10.1002/nbm.70178

**Published:** 2025-11-14

**Authors:** Nada Kamona, Mahdie Hosseini, Michael C. Langham, Felix W. Wehrli

**Affiliations:** ^1^ Department of Radiology, Perelman School of Medicine University of Pennsylvania Philadelphia Pennsylvania USA; ^2^ Department of Bioengineering, School of Engineering and Applied Sciences University of Pennsylvania Philadelphia Pennsylvania USA

**Keywords:** blood flow, graded hypoxia, kidney, MRI, oxygenation

## Abstract

In early kidney disease, tissue hypoxia occurs due to an imbalance between ATP supply and demand. Whole‐organ renal metabolic rate of oxygen (rMRO_2_) is therefore a potential biomarker for assessing renal function. This study evaluated the sensitivity of a quantitative MRI method to detect within‐subject changes in metabolic parameters during hypoxic gas challenges. Ten healthy adults were imaged at 3 T (5 female, ages 23–53 years) while undergoing mild and moderate hypoxia (P_ET_O_2_ 62 and 52 mmHg, respectively). The utilized MRI sequence simultaneously quantified blood flow rate (BFR) and venous oxygen saturation (SvO_2_) at the left renal vein, yielding, together with arterial oxygen saturation (SaO_2_) obtained by pulse oximetry, whole‐organ rMRO_2_ by invoking Fick's Principle. Repeated‐measures ANOVA was used to test differences in metabolic parameters between baseline and hypoxic conditions. SaO_2_ at baseline was 99% ± 1%, while renal SvO_2_ was 92% ± 3%. During progressive hypoxemia, the drop in SvO_2_ (mild 83% ± 4%, moderate 76% ± 5%, *p* < 0.01) paralleled the drop in SaO_2_ (mild 90% ± 1%, moderate 84% ± 2%), such that the arteriovenous difference in oxygenation (AVDO_2_) was constant when compared to baseline (*p* = 1). Renal BFR did not vary significantly between baseline (410 ± 65 mL/min) and hypoxemic conditions (mild, moderate of 430 ± 56 and 440 ± 48 mL/min, *p* > 0.34). Thus, rMRO_2_ did not significantly change during hypoxemia (baseline, mild, and moderate of 140 ± 50, 180 ± 80, and 170 ± 90 (μmol O_2_/min)/100 g, respectively, *p* = 1). In conclusion, the results demonstrate the method's sensitivity in detecting within‐subject changes in metabolic parameters in response to graded hypoxia. Quantitative MRI oximetry may be a feasible tool to assess and longitudinally monitor early metabolic changes in kidney disease.

## Graphical abstract

This study provides experimental evidence in healthy adults of the kidney's physiological response to short‐term, acute hypoxemia, using non‐invasive a quantitative MRI technique. During hypoxemia, findings suggest preserved renal blood flow, coupled with preservation of the arteriovenous difference in oxygen saturation, subsequently resulting in a maintained whole‐organ renal metabolic rate of oxygen. Quantitative MRI‐oximetry may be a promising tool for advancing clinical understanding of renal oxygenation and for detecting early metabolic changes in kidney disease.

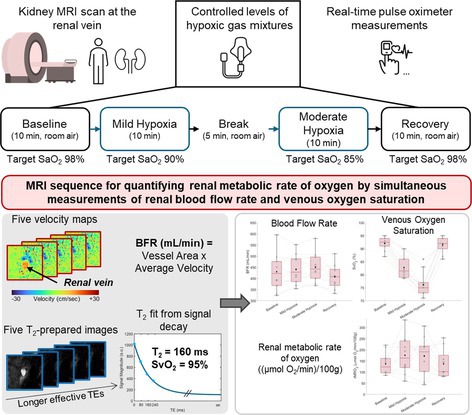

**Abbreviations**
AVDO_2_
: arteriovenous difference in oxygen saturation
BFR: blood flow rate
K‐MOTIVE: Kidney Metabolism of Oxygen via T_2_ and Interleaved Velocity Encoding
rMRO_2_
: renal metabolic rate of oxygen
SaO_2_
: arterial oxygen saturation
SvO_2_
: venous oxygen saturation

## 1 Introduction

Renal metabolic rate of oxygen (rMRO_2_) is a potential physiological biomarker to assess the kidney's metabolic efficiency. In early kidney dysfunction, there is an imbalance between ATP supply and demand [1, 2]. Studies in animal models of diabetes reported elevated rMRO_2_ compared to controls, coupled with no significant differences in renal blood flow rate (BFR) between control and diabetic animals [3, 4, 5, 6]. This elevated rMRO_2_ in the early stages of diabetic chronic kidney disease (CKD) suggests that the increases in renal metabolism are made possible by increased oxygen extraction rather than elevation of renal blood flow. Furthermore, the kidneys are susceptible to tissue hypoxia despite the kidney being one of the most highly perfused organs in the body, receiving 20%–25% of the cardiac output at rest [7]. Kidney hypoxia is believed to be a common pathway for the progression of CKD and acute kidney injury [1, 2, 8, 9, 10]. Thus, rMRO_2_ may be a sensitive indicator of the progression of renal metabolic dysfunction prior to the onset of structural damage.

MRI is the only imaging modality that can noninvasively quantify rMRO_2_ in vivo. Blood oxygenation level–dependent (BOLD) MRI signal, expressed as R_2_* (i.e., 1/T_2_*, the effective transverse relaxation rate), increases with deoxyhemoglobin concentration, and thus may serve as an indirect indicator of renal tissue oxygenation [11, 12, 13]. In CKD, studies have shown that R_2_* in the renal medulla and cortex correlated with estimated glomerular filtration and renal function decline [11, 14, 15]. However, R_2_* cannot be expressed in physiological units, as it only provides relative oxygenation changes. Furthermore, R_2_* measurements can be confounded by other factors such as blood volume fraction, hematocrit, and renal blood pressure [16, 17].

One can quantify whole‐organ rMRO_2_ in vivo using MRI‐based oximetry by exploiting Fick's Principle, requiring measurements of venous oxygen saturation (SvO_2_) and BFR. Prior work in the authors' laboratory developed a non‐invasive and rapid pulse sequence called K‐MOTIVE (Kidney Metabolism of Oxygen via T_2_ and Interleaved Velocity Encoding) that simultaneously measures SvO_2_ and BFR at the draining vein of the kidney [18, 19]. Given the subject's hematocrit and kidney mass, whole‐organ rMRO_2_ can then be calculated in physiological units of moles of oxygen per minute per gram of tissue. K‐MOTIVE has recently been evaluated in healthy adults, yielding reproducible and physiologically plausible metabolic parameters [18, 19, 20].

The primary goal of this study is to evaluate the sensitivity of the K‐MOTIVE method to detecting changes in renal hemodynamics and rMRO_2_ in response to graded hypoxia. A secondary objective is to characterize the renal physiological response in humans to transient hypoxemic episodes, thereby partially mimicking the pathophysiology of kidney hypoxia during early‐stage CKD. During a hypoxic gas challenge, it is hypothesized that renal SvO_2_ will decrease in parallel with the reduction in arterial oxygen saturation (SaO_2_), with renal BFR remaining invariant [21, 22, 23], resulting in preserved rMRO_2_ relative to baseline. In contrast, animal studies suggest early‐stage diabetic CKD may be characterized by increased oxygen extraction (i.e., reduced SvO_2_) and normal BFR, to compensate for the increased workload (and thus increased rMRO_2_) [3, 4, 5, 6]. To the best of the authors' knowledge, this may be the first study in humans to use MRI to quantitatively assess rMRO_2_ in response to hypoxic gas challenges.

## 2 Materials and Methods

### 2.1 rMRO_2_ Exploiting Conservation of Mass Principle

Renal metabolic rate of oxygen (rMRO_2_) can be derived using Fick's Principle:

(1) $${\text{rMRO}}_{2}=\left(\frac{C_{\mathrm{RBC}}\times \mathrm{Hct}}{\text{kidney mass}}\right)\times \mathrm{BFR}\times \left({\mathrm{SaO}}_{2}-{\mathrm{SvO}}_{2}\right)$$

where $C_{\mathrm{RBC}}$ is the oxygen carrying capacity of red blood cells (19.93 μmol O_2_/mL RBC [24]), Hct is the subject‐specific hematocrit, BFR is blood flow rate (mL/min), SaO_2_ is arterial oxygen saturation, and SvO_2_ is venous oxygen saturation. By measuring BFR and SvO_2_ at an imaging slice perpendicular to the renal vessels, rMRO_2_ of a single kidney can be estimated in units of (μmol O_2_/min)/100 g [18]. SaO_2_ can be measured with a pulse oximeter or assumed to be 98% for healthy adults. Hematocrit is measured with a standard finger prick while kidney mass is determined from an anatomical kidney MRI scan and a known tissue density [25].

In addition to quantifying rMRO_2_ of a single kidney, one can indirectly quantify the bilateral rMRO_2_ of the left and right kidneys by concurrently measuring SvO_2_ and BFR at the suprarenal and infrarenal inferior vena cava (IVC) [19]. The bilateral approach, representing the total metabolism of the two kidneys normalized by total mass, is possible because the difference in BFR between the suprarenal and infrarenal IVC should equal the total venous outflow from the renal veins. As such, the Fick's Principle equation is modified to estimate the bilateral rMRO_2_ as follows [19]:

(2) $$\begin{array}{c} \text{bilateral}\;{\text{rMRO}}_{2}=\left(\frac{C_{\mathrm{RBC}}\times \mathrm{hct}}{\text{total kidney mass}}\right)\times \left(Q_{V}^{s}-Q_{V}^{i}\right)\\ \;\times \left({\mathrm{SaO}}_{2}-\left(\frac{\left(Q_{V}^{s}\cdot {\mathrm{SvO}}_{2}^{s}\right)-\left(Q_{V}^{i}\cdot {\mathrm{SvO}}_{2}^{i}\right)}{Q_{V}^{s}-Q_{V}^{i}}\right)\right) \end{array}$$

where the bilateral SvO_2_ term is modified to be the flow‐weighted average venous oxygenation from both renal veins, while $Q_{V}^{s}$ and $Q_{V}^{i}$ are the venous flow rate terms at the suprarenal and infrarenal IVC, respectively, reflecting total outflow from the left and right renal veins. Equation (2) is further simplified to:

(3) $$\begin{array}{c} \text{bilateral}\;{\text{rMRO}}_{2}=\left(\frac{C_{\mathrm{RBC}}\times \mathrm{hct}}{\text{total kidney mass}}\right)\\ \times \left(Q_{V}^{s}\left({\mathrm{SaO}}_{2}-{\mathrm{SvO}}_{2}^{s}\right)-Q_{V}^{i}\left({\mathrm{SaO}}_{2}-{\mathrm{SvO}}_{2}^{i}\right)\right) \end{array}$$

Equations (1) and (3) were used for computing the individual and bilateral rMRO_2_, respectively. In both equations, the difference between SaO_2_ and SvO_2_ will be referred to as the arteriovenous difference in oxygen saturation (AVDO_2_). Full justification and derivation for the bilateral rMRO_2_ approach is discussed in Deshpande et al. [19].

### 2.2 MRI Pulse Sequence for Quantifying rMRO_2_

The K‐MOTIVE sequence was detailed in prior work [18, 19, 20]. Briefly, the sequence simultaneously measures BFR and transverse relaxation time (T_2_) of blood water in 22 s. The sequence interleaves a spiral‐readout phase‐contrast (PC), global T_2_‐preparation, and a golden‐angle radial balanced steady‐state free precession (bSSFP) readout (Figure S1). Approximately every 4.4 s, the sequence acquires one velocity map and one T_2_‐prepared image. Thus, five velocity maps and five T_2_‐prepared images are acquired in one K‐MOTIVE acquisition. The average signal intensity in the T_2_‐prepared images is used for T_2_ decay fit of blood water, which in turn is converted to SvO_2_ using a calibration model [26, 27]. Sequence parameters (Table S1) were previously reported in Kamona et al. [20].

### 2.3 Experimental Procedure

To examine the sensitivity of the proposed K‐MOTIVE sequence to within‐subject changes in metabolic parameters, 10 healthy participants (5 female, ages 30 ± 9 years, range 23–53 years) were recruited to undergo a hypoxic gas challenge. Participants were scanned at 3 T (Prisma, Siemens, Erlangen, Germany) using 18‐channel body‐array and 32‐channel spine coils. All MR imaging was performed after obtaining informed consent, in accordance with the authors' Institutional Review Board requirements.

Each participant was fitted with a specialized gas breathing mask and tightly sealed with medical skin tape. Gas delivery was controlled by an MRI‐compatible, computer‐controlled, sequential gas delivery circuit (RespirAct RA‐MR system, Thornhill Research Inc., Toronto, Canada) that targets arterial partial pressure of oxygen (PaO_2_) and carbon dioxide (PaCO_2_) independently of tidal volumes and breathing frequency, and monitors real‐time end‐tidal partial pressure of oxygen (P_ET_O_2_) and carbon dioxide (P_ET_CO_2_). Mild and moderate isocapnic (P_ET_CO_2_ target 40 mmHg) hypoxia levels were achieved at P_ET_O_2_ of 62 mmHg (equivalent to an FiO_2_ ~ 13%) and 52 mmHg (FiO_2_ ~ 11%), and equivalent to a target SaO_2_ of approximately 90% and 85%, respectively [28, 29]. SaO_2_ was monitored throughout the scan using an MRI‐compatible pulse oximeter (Nonin, Model 7500FO, Plymouth, MN, USA). Prior to the MRI scan, participants were exposed to the hypoxic gas stimulus outside the scanner to ensure they could tolerate it for several minutes.

**FIGURE 1:**
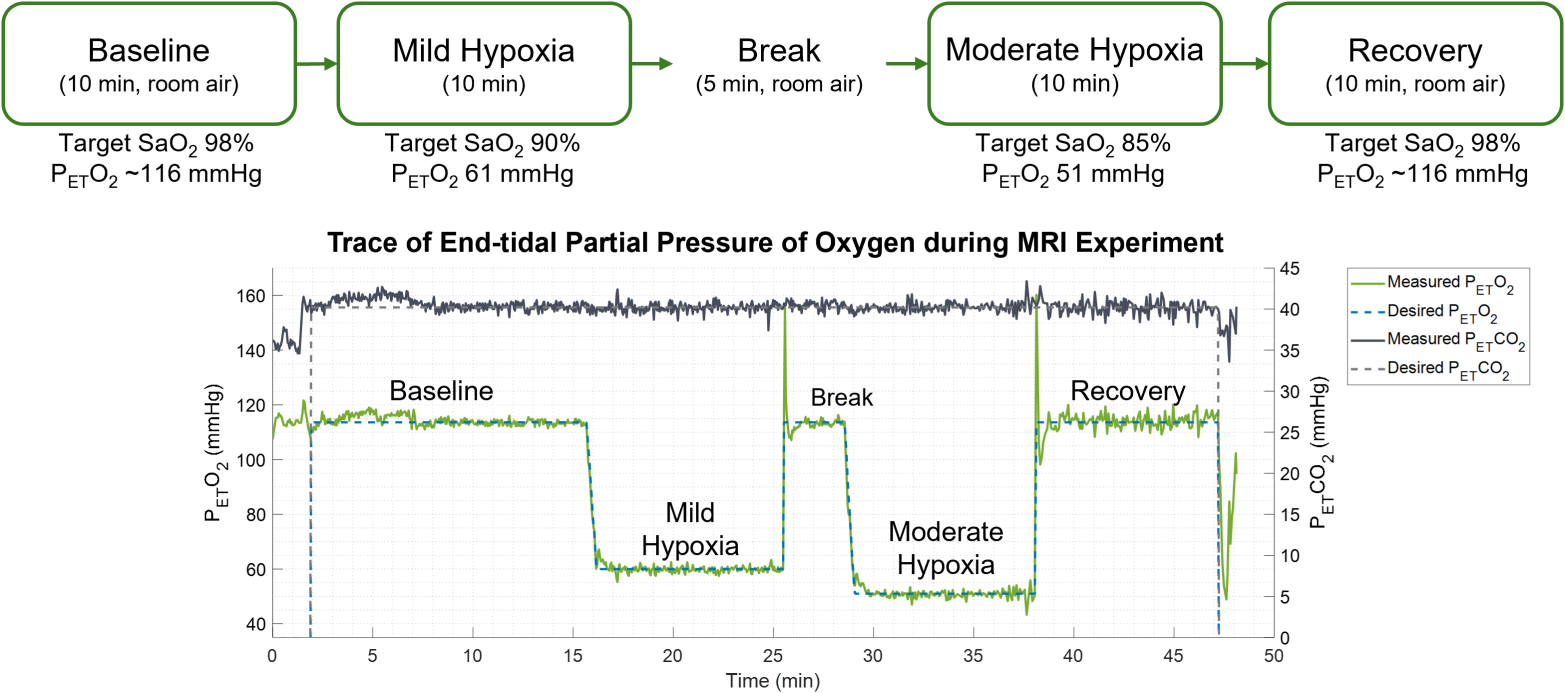
Experimental procedure of the isocapnic hypoxic gas challenge, consisting of four respiratory stages at baseline, mild hypoxia, moderate hypoxia, and recovery. Baseline and recovery stages refer to breathing normoxic/normocapnic air (i.e., room air). The bottom figure is an example real‐time trace of P_ET_O_2_ and P_ET_CO_2_ measurements throughout the MRI experiment in a healthy 25‐year‐old female, illustrating how the participant met the target values.

Participants were imaged during each of the following breathing conditions (Figure 1): baseline, mild hypoxia, moderate hypoxia, and recovery, with each stage lasting approximately 10 min. Baseline and recovery stages refer to normoxic/normocapnic air (i.e., room air). At the beginning of each hypoxia level, participants were given one to 2 min to reach a stable target SaO_2_ before MR imaging. The two hypoxia stages were separated by a 5‐min period of breathing room air to increase subject comfort.

**FIGURE 2:**
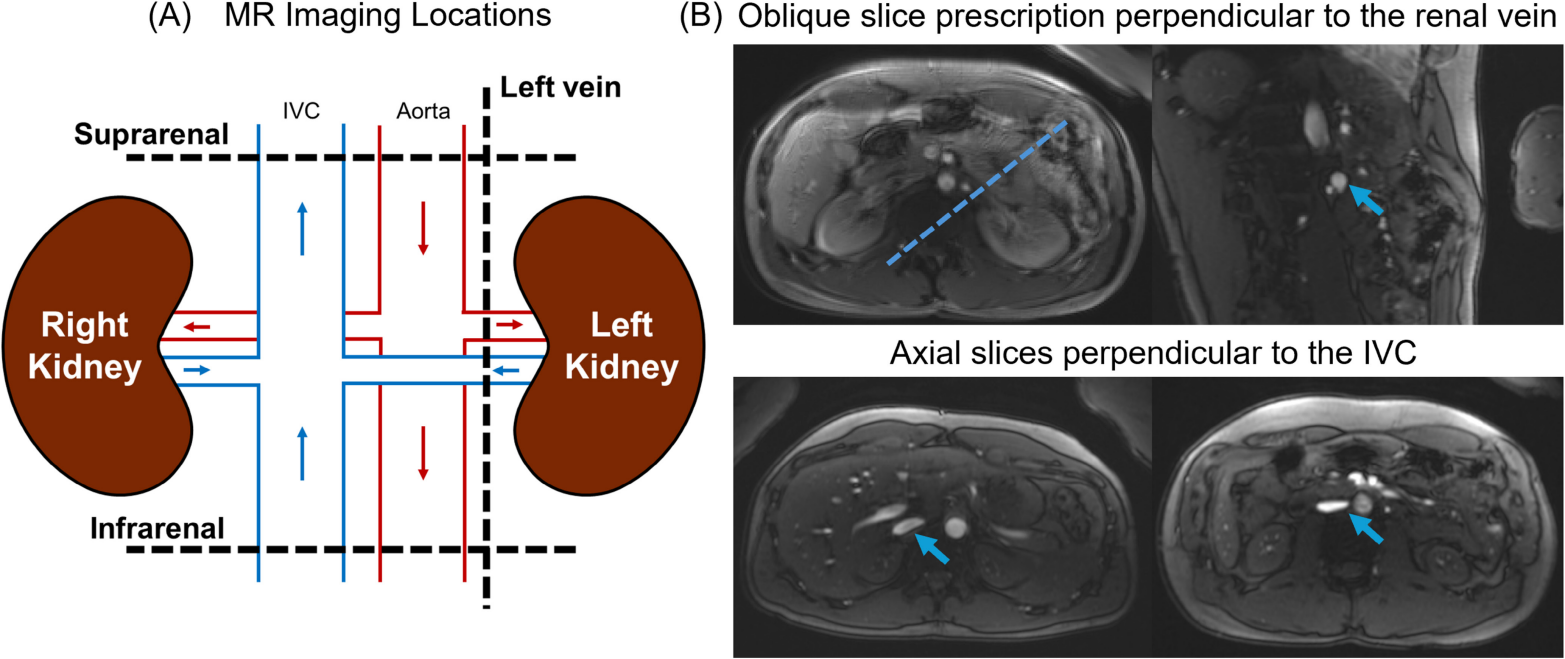
MR imaging slice locations for quantification of whole‐organ rMRO_2_ in vivo. (**A)** Schematic of the kidneys, aorta and IVC. Two‐dimensional slices were prescribed along the dashed black lines. (**B)** Example localizer time‐of‐flight images from 32‐year‐old male. An oblique slice was selected perpendicular to the renal vein (blue line and arrow), while axial slices were selected perpendicular to the suprarenal and infrarenal IVC (blue arrows).

Participants were imaged with K‐MOTIVE at the left renal vein, suprarenal IVC, and infrarenal IVC. Five to six free‐breathing K‐MOTIVE measurements were acquired at each breathing condition and imaging location, with each measurement lasting 22 s (i.e., five measurements totaling approximately 2 min). Prior to K‐MOTIVE acquisition and while the participant breathed room air, a breath‐hold time‐of‐flight localizer sequence was utilized to identify imaging slices perpendicular to the left renal vein and the IVC (Figure 2). An additional 20‐s breath‐hold gradient‐echo sequence was performed to obtain subject‐specific kidney anatomical images. K‐MOTIVE was not acquired at the right renal vein due to time constraints and to limit the hypoxic gas exposure to no more than 10 min per stage.

### 2.4 Image Processing & Data Analysis

K‐MOTIVE T_2_‐prepared images were reconstructed offline in MATLAB R2023a (MathWorks, Natick, MA, USA) using a nonuniform fast Fourier Transform [30]. PC images were reconstructed with SENSE using SigPy to remove signal aliasing outside the desired FOV (if any) [31, 32, 33]. Further reconstruction details were previously reported in Kamona et al. [20].

Regions of interest (ROIs) were segmented from the T_2_‐prepared bSSFP images and PC velocity maps using a semi‐automated approach. Initial segmentations were derived from two U‐Net deep‐learning models [34], trained separately for each K‐MOTIVE image type using previously collected data. Additional information on model training and performance metrics is provided in the Supporting Information. To minimize the impact of segmentation errors on the study outcomes, all automatically generated ROIs from the U‐Net models were visually examined, and where needed, the segmentations were manually edited for any observed errors. The average signal intensity within the ROI was used to fit the T_2_ decay curve from the bSSFP images, which is then converted to SvO_2_ using a calibration model [26, 27]. From each PC image, the average velocity in the ROI was multiplied by the ROI area to calculate BFR. The final BFR value was averaged across the five velocity maps obtained from one K‐MOTIVE acquisition. Subject‐specific kidney mass was calculated by manually segmenting the kidney anatomic images and multiplying the resulting kidney volume by the known tissue density of 1.06 g/mL [25].

Repeated‐measures (RM) ANOVA was used to determine the effect of breathing hypoxic gas on metabolic parameters (T_2_, SvO_2_, AVDO_2_, BFR, rMRO_2_) at each imaging location (left renal vein, suprarenal and infrarenal IVC). RM‐ANOVA was further used to compare differences between the unilateral rMRO_2_ of the left kidney and the indirect bilateral rMRO_2_ at each hypoxia level. Post hoc pairwise comparisons were performed using Bonferroni's method to account for multiple comparisons. The *p*‐values were adjusted by multiplying each uncorrected *p*‐value by the number of comparisons to maintain the *p*‐values' significance threshold at 0.05. Statistical analysis was performed in R version 4.3.2 (R Foundation for Statistical Computing, Vienna, Austria).

## 3 Results

Ten healthy participants underwent a kidney MRI scan while breathing a graded hypoxic gas mixture. Due to scanner technical difficulties, one subject was not scanned during the recovery stage. Consequently, data from this participant was included in descriptive summaries but excluded from RM‐ANOVA analyses. Full participant characteristics are given in Table 1. All participants tolerated the isocapnic hypoxic gas stimulus well, with most reporting only deeper and more rapid breathing during moderate hypoxia. Figure 1 illustrates graded hypoxia stages along with example real‐time traces of P_ET_O_2_ and P_ET_CO_2_ measurements throughout the MRI experiment in a healthy participant using the RespirAct gas control system.

**TABLE 1:** Participant demographics, kidney mass, and hematocrit.

Parameter	Value^α^
Number of participants	10 (5 M, 5 F)
Age (years)	30 ± 9 (23–53)
Height (m)	1.71 ± 0.12 (1.50–1.91)
Weight (kg)	71 ± 14 (55–100)
Body mass index (kg/m^2^)	24 ± 3 (21–30)
Left kidney mass (g)	172 ± 29 (124–226)
Right kidney mass (g)	159 ± 26 (117–221)
Hematocrit (%)^β^	42 ± 4 (34–46)

*Note:* ^α^ Values are expressed as mean ± standard deviation (range), where applicable.

^β^ Hemoglobin (g/dL) measurement was performed via a standard finger prick test using HemoCue Hb 201+/801 systems (HemoCue America, Brea, CA, USA), converted to hematocrit using the equation Hct(%) = Hb (g/dL) /0.34.

**FIGURE 3:**
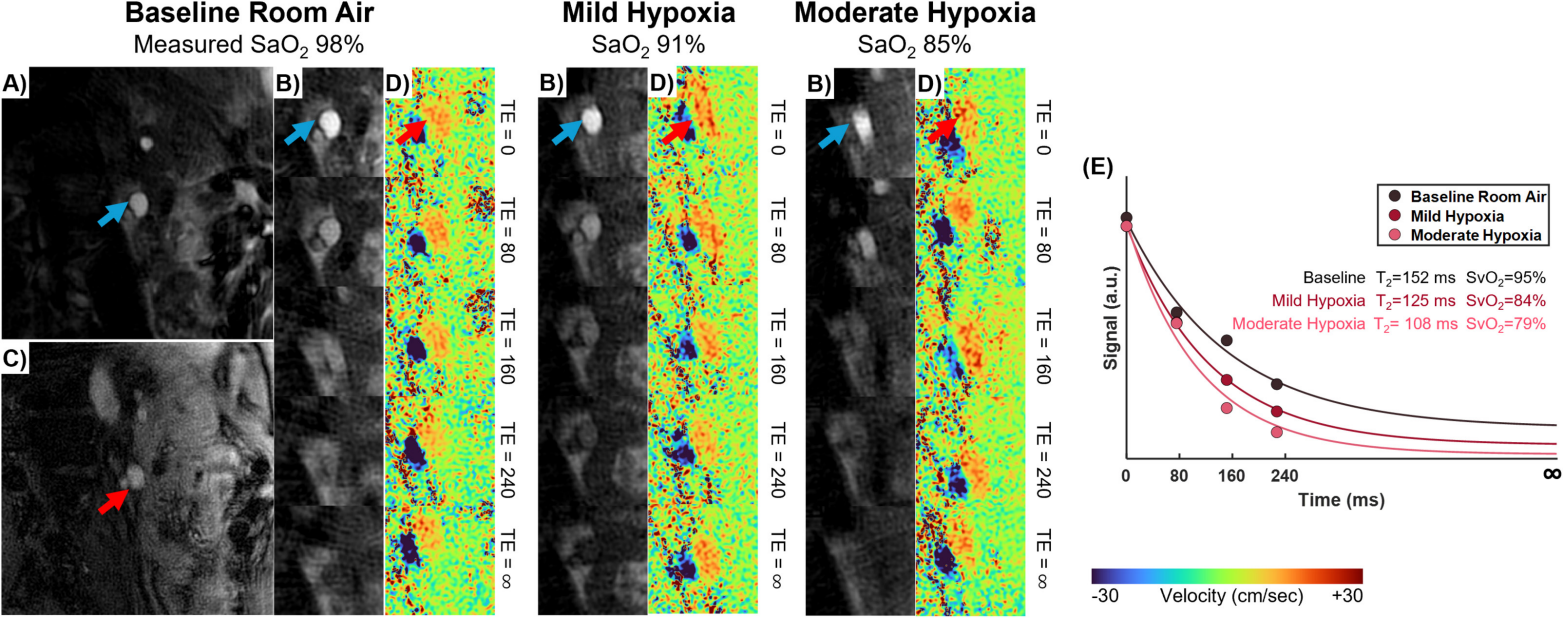
Example free‐breathing K‐MOTIVE outputs at the left renal vein in one healthy adult (32‐year‐old male). K‐MOTIVE outputs two sets of images: five background‐suppressed T_2_‐prepared bSSFP images and five PC velocity maps. (**A)** Background‐suppressed T_2_‐prepared bSSFP image at an effective TE of 0 ms. (**B)** Magnified views of T_2_‐prepared bSSFP images at five increasing effective TEs. (**C)** Magnitude image from PC module at first TE. (**D)** Corresponding magnified velocity maps from the PC module acquired across five interleaves of the sequence. Panels (**B)** and (**D)** are shown for all breathing conditions at baseline, mild and moderate hypoxia. (**E)** T_2_‐fit from the signal decay obtained from five T_2_‐prepared images, shown for data acquired while the participant breathed baseline, mild and moderate hypoxic gas. During hypoxemia, faster signal decay commensurate higher deoxyhemoglobin content in the blood. However, renal venous BFR did not change during hypoxemia.

**FIGURE 4:**
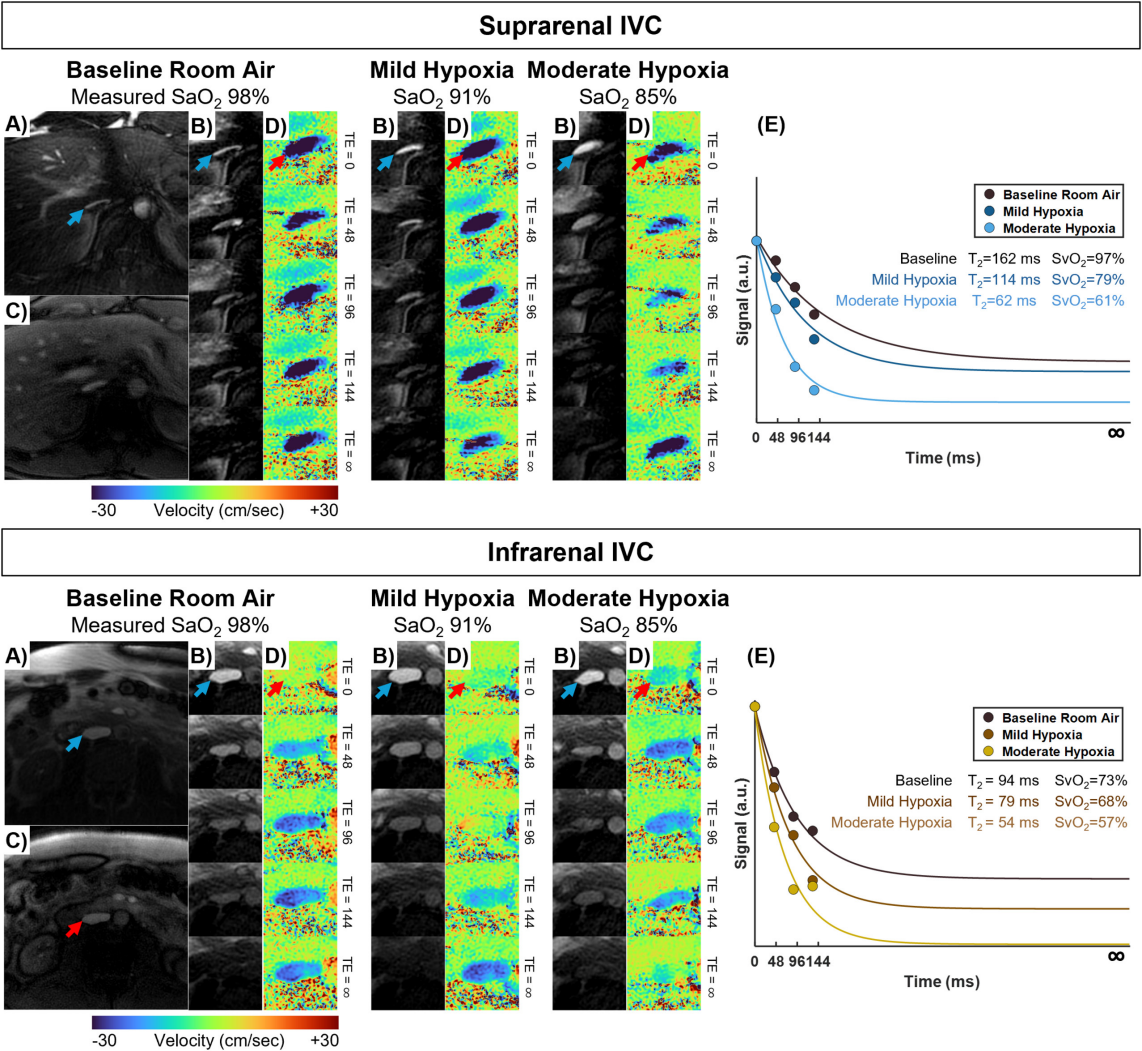
Example K‐MOTIVE outputs at the suprarenal and infrarenal IVC in one healthy adult (32‐year‐old male). (**A)** Background‐suppressed T_2_‐prepared bSSFP image at an effective TE of 0 ms. (**B)** Magnified views of T_2_‐prepared bSSFP images at five effective TEs. (**C)** Magnitude image from PC module at first TE. (**D)** Corresponding magnified velocity maps from the PC module acquired across five interleaves of the sequence. Panels (**B)** and (**D)** are shown for all breathing conditions at baseline, mild and moderate hypoxia. (**E)** T_2_‐fit from the signal decay obtained from five T_2_‐prepared images, shown for data acquired while the participant breathed baseline room air, mild and moderate hypoxic gas. During hypoxemia, faster signal decay commensurate higher deoxyhemoglobin content in the blood.

**FIGURE 5:**
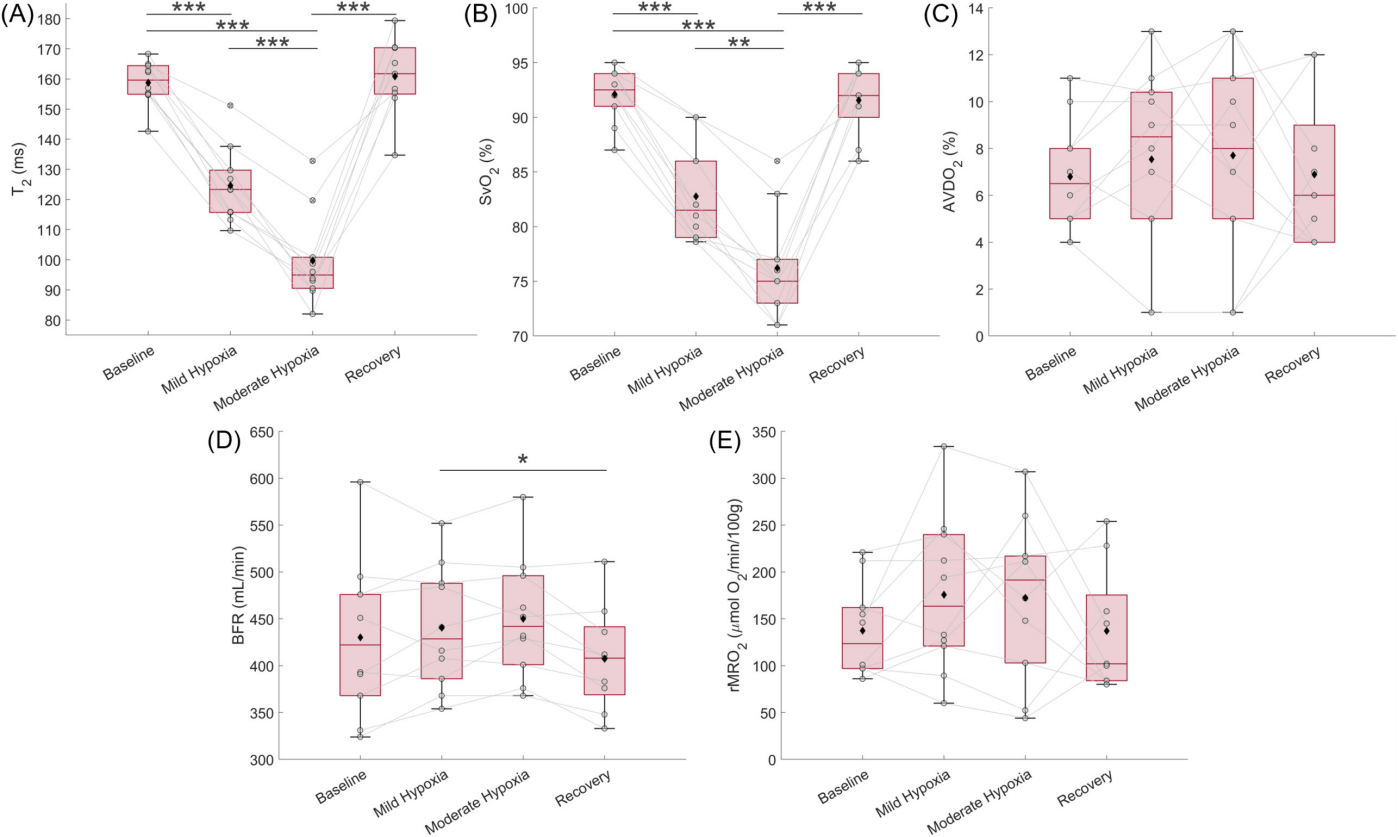
Left kidney metabolic parameters for 10 healthy participants at four respiratory conditions: baseline room air, mild hypoxia, moderate hypoxia, and recovery at room air. (**A)** T_2_ of blood water. (**B)** SvO_2_. (**C)** AVDO_2_. (**D)** BFR. (**E)** rMRO_2_. The boxplot outlines are the 25th, 50th, and 75th percentiles, the whiskers are the non‐outlier data range, the black diamonds are average values, the cross shapes are outliers, the gray circles and lines are individual subjects. RM ANOVA was used to test within‐subject differences across respiratory conditions, with *p < 0.05, **p < 0.01, ***p < 0.001. Abbreviations: SvO_2_, venous oxygen saturation; AVDO_2_, arteriovenous oxygen difference; BFR, blood flow rate; rMRO_2_, renal metabolic rate of oxygen.

**FIGURE 6:**
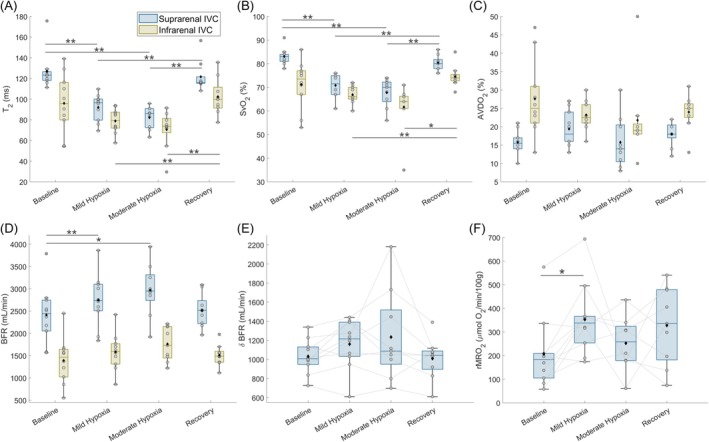
Summary of IVC metabolic parameters for 10 healthy participants at four respiratory conditions: baseline room air, mild hypoxia, moderate hypoxia, and recovery at room air. (**A)** T_2_ of blood water. (**B)** SvO_2_. (**C)** AVDO_2_. (**D)** BFR. (**E)** BFR difference between the suprarenal and infrarenal locations. (**F)** indirect bilateral rMRO_2_. The boxplot outlines are the 25^th^, 50^th^, and 75^th^ percentiles, the whiskers are the non‐outlier data range, the black diamonds are average values, the cross shapes are outliers, the gray circles and lines are individual subjects. RM ANOVA was used to test within‐subject differences across respiratory conditions, with *p < 0.05, **p < 0.01. Abbreviations: SvO_2_, venous oxygen saturation; AVDO_2_, arteriovenous oxygen difference; BFR, blood flow rate; rMRO_2_, renal metabolic rate of oxygen.

An overview of slice locations for quantification of whole‐organ rMRO_2_ is in Figure 2. Example free‐breathing K‐MOTIVE images and T_2_‐decay fit curves across breathing conditions are shown in Figure 3 (left renal vein) and Figure 4 (suprarenal and infrarenal IVC). A summary of metabolic parameters for all imaging locations and breathing conditions is provided in Table 2 and Figures 5 and 6. Results from RM‐ANOVA and post hoc pairwise comparisons are summarized in Table S2 and Table 3, respectively.

**TABLE 2:** Summary of all measured metabolic parameters in 10 healthy participants, imaged during four breathing conditions at baseline (room air), mild hypoxia, moderate hypoxia, and recovery (room air).

		Breathing condition and target SaO_2_			
	**Measured parameters**	**Baseline** **98%**	**Mild hypoxia 90%**	**Moderate hypoxia** **85%**	**Recovery** **98%**
Peripheral via pulse oximeter	SaO_2_ (%)	99 ± 1	90 ± 1	84 ± 2	98 ± 1
Left kidney	SvO_2_ (%)	92 ± 3	83 ± 4	76 ± 5	92 ± 3
	AVDO_2_ (%)	7 ± 2	8 ± 4	8 ± 4	7 ± 3
	T_2_ (ms)	159 ± 7	125 ± 13	100 ± 15	161 ± 13
	BFR (mL/min)^α^	410 ± 65	430 ± 56	440 ± 48	410 ± 56
	rMRO_2_ ((μmol O_2_/min)/100 g)^α^	140 ± 50	180 ± 80	170 ± 90	140 ± 70
Suprarenal IVC	SvO_2_ (%)	83 ± 4	71 ± 5	68 ± 6	80 ± 3
	AVDO_2_ (%)	16 ± 3	19 ± 5	16 ± 7	18 ± 3
	T_2_ (ms)	127 ± 18	92 ± 13	82 ± 11	122 ± 15
	BFR (mL/min)^α^	2420 ± 650	2750 ± 560	2970 ± 590	2510 ± 380
Infrarenal IVC	SvO_2_ (%)	71 ± 10	67 ± 4	62 ± 10	75 ± 5
	AVDO_2_ (%)	28 ± 10	23 ± 4	22 ± 11	24 ± 5
	T_2_ (ms)	96 ± 29	79 ± 12	71 ± 18	102 ± 18
	BFR (mL/min)^α^	1390 ± 530	1580 ± 420	1760 ± 380	1510 ± 240
Bilateral (Suprarenal—infrarenal IVC)^γ^	∆ BFR^γ^	1030 ± 180	1160 ± 250	1230 ± 470	1010 ± 210
	rMRO_2_ ((μmol O_2_/min)/100 g)^α^	210 ± 150	350 ± 150	250 ± 120	330 ± 160

*Note:* ^α^ BFR and rMRO_2_ values were rounded to the tens place.

^γ^ ∆BFR is defined as the BFR difference between the suprarenal and infrarenal IVC.

Abbreviations: AVDO_2_, arteriovenous difference in oxygen saturation (SaO_2_—SvO_2_); BFR, blood flow rate; rMRO_2_, renal metabolic rate of oxygen; SaO_2_, arterial oxygen saturation; SvO_2_, venous oxygen saturation.

**TABLE 3:** *P*‐values from post hoc pairwise comparisons from RM‐ANOVA assessing differences in metabolic parameters between pairs of breathing conditions.

	Pairwise comparison^α^	Baseline vs mild hypoxia	Baseline vs moderate hypoxia	Baseline vs recovery	Mild vs moderate hypoxia	Mild hypoxia vs recovery	Moderate hypoxia vs recovery
Left kidney	T_2_	**< 0.001**	**< 0.001**	1	**< 0.001**	**0.002**	**< 0.001**
	SvO_2_	**< 0.001**	**< 0.001**	1	**0.001**	**0.002**	**< 0.001**
	AVDO_2_	1	1	1	1	1	1
	BFR	0.72	0.34	1	1	**0.03**	0.06
	rMRO_2_	1	1	1	1	1	1
Suprarenal IVC	T_2_	**0.006**	**0.006**	1	1	**0.008**	**0.002**
	SvO_2_	**0.002**	**0.003**	0.89	1	**0.002**	**0.002**
	AVDO_2_	0.49	1	1	0.97	1	1
	BFR	**0.001**	**0.01**	1	0.23	1	0.14
Infrarenal IVC	T_2_	0.41	0.09	1	0.93	**0.006**	**0.004**
	SvO_2_	1	0.44	1	0.91	**0.005**	**0.03**
	AVDO_2_	1	1	1	1	1	1
	BFR	0.14	0.12	1	0.28	1	0.26
Bilateral (Suprarenal – infrarenal IVC)	∆ BFR^β^	0.30	0.86	1	1	0.68	0.63
	rMRO_2_	**0.04**	1	0.72	1	1	1

*Note:* ^α^ RM‐ANOVA was constructed for each metabolic parameter and imaging location. Bonferroni's method was applied to adjust the reported *p*‐values for post hoc multiple comparisons. The *p*‐values were adjusted by multiplying each uncorrected *p*‐value by the number of comparisons, while maintaining the *p*‐values' significance threshold at 0.05.

^β^ The bilateral BFR is defined as the BFR difference between suprarenal and infrarenal IVC.

Abbreviations: AVDO_2_, arteriovenous difference in oxygen saturation (SaO_2_—SvO_2_); BFR, blood flow rate; rMRO_2_, renal metabolic rate of oxygen; SaO_2_, arterial oxygen saturation; SvO_2_, venous oxygen saturation.

Average SaO_2_ across all participants was 99% ± 1% at baseline, dropping to 90% ± 1% and 84% ± 2% during mild and moderate hypoxia, respectively. At the left renal vein, SvO_2_ significantly decreased from baseline to mild hypoxia (92% ± 3%–83% ± 4%, *p* < 0.001), with a further drop from mild to moderate hypoxia levels (83% ± 4%–76% ± 5%, *p* = 0.001). SvO_2_ recovered back to baseline values following breathing hypoxic gas (*p* = 1). Renal BFR (baseline 410 ± 65 mL/min) and AVDO_2_ (baseline 7% ± 2%) did not alter significantly during graded hypoxia when compared to baseline values. As a result, rMRO_2_ of the left kidney remained essentially invariant across all breathing conditions, with a baseline average value of 140 ± 50 (μmol O_2_/min)/100 g.

SvO_2_ of the suprarenal IVC dropped from baseline to mild hypoxia (83% ± 4%–71% ± 5%, *p* = 0.002), mild to moderate hypoxia (71% ± 5%–68% ± 6%, *p* = 0.003), then recovered such that there were no differences between baseline and recovery measurements (*p* = 0.89). On the other hand, SvO_2_ of the infrarenal IVC differed significantly only between the hypoxia and recovery stages (moderate hypoxia 62% ± 10%, recovery 75% ± 5%, *p* = 0.03). The observed decrease in SvO_2_ at the IVC paralleled the decrease in SaO_2_, resulting in no significant change in AVDO_2_ at the suprarenal (baseline 16% ± 3%) and infrarenal IVC (baseline 28% ± 10%) across all breathing conditions (*p* > 0.49).

BFR of the suprarenal IVC increased during the two hypoxia levels (baseline 2420 ± 650 mL/min, moderate hypoxia 2970 ± 590 mL/min, *p* = 0.01), while BFR of the infrarenal IVC trended towards higher values during hypoxia (baseline 1390 ± 530 mL/min, moderate hypoxia 1760 ± 380 mL/min, *p* = 0.12). However, the BFR difference between the two IVC locations (baseline 1030 ± 180 mL/min) did not vary significantly throughout the hypoxic challenges (*p* > 0.30).

When combining SvO_2_ and BFR parameters at the IVC (Equation 3), bilateral rMRO_2_ increased during mild hypoxia (from 210 ± 150 to 350 ± 150 [μmol O_2_/min]/100 g, *p* = 0.04). However, bilateral rMRO_2_ was not different between baseline and moderate hypoxia (*p* = 1). Bilateral rMRO_2_ was not statistically different between baseline (210 ± 150 [μmol O_2_/min]/100 g) and the recovery stage (330 ± 160 [μmol O_2_/min]/100 g, p = 1) despite the increasing trend. Finally, the left kidney and bilateral rMRO_2_ (i.e., total metabolism normalized by total mass of both kidneys) values were not statistically different across breathing conditions (baseline, p = 1; mild hypoxia, *p* = 0.08; moderate hypoxia, p = 1; recovery *p* = 0.27).

## 4 Discussion

Renal MRO_2_ may be a potential clinical biomarker of kidney function in early stages of disease, i.e., prior to the onset of structural damage [2]. The proposed quantitative MRI method for assessing kidney metabolic rate of oxygen has been designed to estimate individual and bilateral whole‐organ rMRO_2_ by simultaneously measuring renal BFR and SvO_2_ in a single pass [18, 19, 20]. This non‐invasive K‐MOTIVE sequence yields one rMRO_2_ measurement in 22 s, and hence, can allow successive free‐breathing repeat measurements to alleviate motion without significantly increasing overall scan time. The present study evaluated the sensitivity of K‐MOTIVE to detect within‐subject changes in metabolic parameters during exposure to a graded hypoxic gas mixture inside the MRI scanner. Furthermore, the results provide experimental insights into human renal physiology in response to short‐term, acute hypoxemic episodes, which may shed light on the pathophysiology in CKD patients.

Baseline metabolic parameters were physiologically plausible and in agreement with prior studies. The average peripheral SaO_2_ was 99% ± 1% paired with a renal SvO_2_ of 92% ± 3%, consistent with previously reported renal SvO_2_ values of 87%–93% [18, 19, 20, 35, 36]. Other studies estimate BFR at approximately 400–500 mL/min for the left renal vein [37, 38], 2000–2500 mL/min for the suprarenal IVC [39], and 1200–1400 mL/min for infrarenal IVC [39, 40]. These values compare favorably with baseline BFR of the renal vein and IVC in the present work. Additionally, left rMRO_2_ was 140 ± 50 (μmol O_2_/min)/100 g, while the bilateral rMRO_2_ had a baseline average of 210 ± 150 (μmol O_2_/min)/100 g, both consistent with prior values of 140–350 (μmol O_2_/min)/100 g [18, 19, 20, 36, 41, 42].

Renal vein oxygen saturation dropped during hypoxemia while maintaining the same baseline arteriovenous difference in oxygen saturation. In De Keijzer et al.'s work [35], designed to validate a near‐infrared device for quantifying regional oxygen metabolism in the kidneys, healthy adults breathed a controlled hypoxic gas mixture at five steps while blood samples were obtained via renal vein venipuncture. At target SaO_2_ of 90% and 85%, renal vein SvO_2_ reached 81.7% ± 2.8% and 76.6% ± 2.9%, respectively, closely matching MRI‐derived values obtained in the present study of 83% ± 4% and 76% ± 5% at the left renal vein. Furthermore, because renal BFR and AVDO_2_ (i.e., the difference between SaO_2_ and SvO_2_) were preserved during the hypoxic gas stimulus, average left rMRO_2_ remained virtually unaltered at 140–180 (μmol O_2_/min)/100 g. Similarly, renal oxygen consumption in animal studies was found to be relatively unaffected during hypoxemia compared to normoxic conditions [21, 23, 43]. It is noted here that a few participants had elevated rMRO_2_ during the hypoxia stimulus due to an increase in AVDO_2_ compared to their baseline values.

Hypoxemia did not alter venous renal blood flow, suggesting the absence of a whole‐organ hyperemic response. Under normal physiological conditions, renal blood flow regulation is more complex than that of other organs where metabolic demand is usually the primary determinant. In the brain, for instance, vasodilation occurs in response to hypoxemia so as to increase blood flow and subsequently oxygen delivery to the tissue [44, 45]. However, the vasodilatory response in the kidney seems to be relatively blunted when compared to other organs [21, 22, 46] with studies showing variable effects of hypoxemia on renal blood flow. Some studies revealed that hypoxemia may induce a renal sympathetic response in kidneys with intact renal nerves, resulting in renal vasoconstriction and lower BFR [47, 48, 49]. In other reports, anesthetized animals that breathed hypoxic gas mixtures did not induce a clear change in renal BFR [21, 22, 23]. If the kidneys were to respond to hypoxemia by increasing blood flow, the resulting rise in glomerular filtration would drive up oxygen consumption [1, 2]. While preserving (or decreasing) BFR during hypoxemia may be counterintuitive, it allows the kidney to preserve its major functions, such as filtration of blood plasma, maintenance of extracellular fluid volume, and regulation of erythrocyte production [22, 46]. This adaptive mechanism, that prioritizes functional requirements over local metabolic needs, might render the kidney more susceptible to hypoxic injury [22, 46].

The proposed hypoxic gas challenge in healthy adults may partially mimic the hypothesized pathophysiology of early‐stage diabetic CKD. Several studies found elevated rMRO_2_ in rat models of diabetes compared to control groups, coupled with no significant changes in renal BFR, suggesting that increased oxygen extraction (i.e., reflected by reduced SvO_2_) serves to meet the heightened kidney workload [3, 4, 5, 6]. In the present study, healthy adults had lower renal SvO_2_ and unaltered BFR during graded hypoxia, paralleling observations in animal models of diabetic CKD. However, kidney workload should be invariant during hypoxemia given that all participants are healthy, which was indeed reflected in the unaltered AVDO_2_ and rMRO_2_ measurements.

The indirect bilateral rMRO_2_ was statistically comparable to the unilateral rMRO_2_ at the left kidney despite the former having higher values on average. The bilateral rMRO_2_ is hypothesized to represent the total metabolism of the left and right kidneys normalized by total mass. Since participants in this study are healthy adults with no prior kidney disease, the left and right kidneys are assumed to have equivalent rMRO_2_, and hence, the bilateral rMRO_2_ and left rMRO_2_ should also be approximately equal. The discrepancy between the two approaches, which is most evident at the mild hypoxia and recovery stages, is attributed to error propagation from uncertainties in measurements of BFR and SvO_2_. Error propagation analysis shows that the Fick's Principle equation is sensitive to measurement errors in SvO_2_. Full details of the error propagation analysis can be found in prior studies [18, 19, 20]. Nevertheless, rMRO_2_ values from the unilateral and bilateral approach may still be physiologically plausible [19, 20, 36, 41, 42]. Future studies should validate MRI‐based rMRO_2_ values against clinical standards (e.g., blood gas analysis from blood samples) or in animals.

Renal MRO_2_, along with SvO_2_ and BFR, may be potential biomarkers for the diagnosis and monitoring of kidney disease [1, 2, 3, 4]. Although clinical validation is still required, K‐MOTIVE‐based metabolic parameters may be valuable in both symptomatic and asymptomatic patients, primarily the latter for early detection of kidney disease. This includes at‐risk populations such as diabetic patients, where early intervention is crucial. Beyond diagnosis, K‐MOTIVE's ability to non‐invasively detect within‐subject changes, as demonstrated in the hypoxia experiments, suggests potential utility for longitudinal monitoring in clinical settings and drug trials.

Quantitative MRI methods for measuring rMRO_2_ are few, underscoring an unmet need in the field. Recently, Xu et al. [50] introduced a T_2_‐based MRI sequence, termed TRUFIFA, that measures whole‐organ SvO_2_ at the renal vein in 4 min under free‐breathing conditions. However, unlike K‐MOTIVE, which quantifies whole‐organ rMRO_2_ by simultaneously measuring both SvO_2_ and BFR, TRUFIFA measures only T_2_ of venous blood, thus requiring a separate PC sequence to determine renal BFR. In addition to whole‐organ oximetry techniques, quantitative BOLD can generate parametric maps of renal oxygen saturation of the cortex and medulla in physiological units [41, 51]. In the first and only quantitative MRI study in CKD patients to date, Prasad et al. [51] reported that late‐stage CKD patients exhibited lower renal oxygen saturation and blood partial pressure of oxygen compared to healthy matched controls in both the cortex and medulla, despite insignificant differences in R_2_* values between the two cohorts. However, this approach requires a lengthy protocol and the use of a contrast agent to determine regional blood volume fraction. Finally, it is yet to be demonstrated that renal metabolic parameters can serve as diagnostic biomarkers in the early stages of CKD.

This study has several limitations. First, measurement uncertainty in BFR and SvO_2_ magnifies the uncertainty in rMRO_2_ quantification. Larger errors are expected for the bilateral rMRO_2_ approach because more parameters must be measured (Equation 3) at two imaging locations, which may explain the discrepancies between the unilateral and bilateral rMRO_2_ values. Second, measured blood oxygenation levels during the hypoxic challenges were not validated via catheterization to avoid exposing participants to invasive and potentially risky procedures. Third, K‐MOTIVE estimates whole‐organ rMRO_2_, and thus, regional differences between the medulla and cortex cannot be differentiated. Nevertheless, whole‐organ rMRO_2_ may still reflect metabolic changes in the early stages of disease [3, 4, 5]. Fourth, although free‐breathing K‐MOTIVE was shown to yield data comparable to breath‐holding in prior work [20], participants exposed to moderate hypoxia reported breathing more deeply and rapidly, which may have increased motion artifacts in some cases. When severe motion artifacts occurred, images were discarded without impacting the number of available acquisitions used in the final analysis, given that five to six K‐MOTIVE repetitions were collected per breathing condition for each participant.

In conclusion, the proposed quantitative MRI method enables noninvasive, rapid quantification of rMRO_2_ by simultaneously measuring BFR and SvO_2_ at the renal vein or at the IVC. Further, the MRI method was shown to be able to detect within‐subject changes in response to graded hypoxia, demonstrating its feasibility for disease diagnosis and longitudinal patient monitoring. To the best of the authors' knowledge, this represents the first MRI‐based study in humans to quantitatively assess renal vascular‐metabolic parameters in response to a hypoxic gas stimulus.

## Author Contributions

F.W.W. and M.C.L. conceived and designed research. N.K. and M.H. performed experiments and analyzed data. F.W.W., M.C.L., and N.K. interpreted the results. N.K. drafted the manuscript. All authors edited, revised, and approved the final version of the manuscript.

## Conflicts of Interest

The authors declare no conflicts of interest.

## Supporting information


**Figure S1:** Overview of the K‐MOTIVE MRI method (Kidney Metabolism of Oxygen via T_2_ and Interleaved Velocity Encoding), designed to noninvasively estimate renal metabolic rate of oxygen (rMRO_2_) in vivo.
**Figure S2:** Example segmentation of five K‐MOTIVE PC images overlayed on the corresponding magnitude images, demonstrating high agreement with the ground‐truth manual segmentation.
**Figure S3:** Example segmentation of five K‐MOTIVE T_2_‐prepared bSSFP images overlayed on the corresponding images, demonstrating high agreement with the ground‐truth manual segmentation.
**Figure S4:** Example segmentation of five K‐MOTIVE T_2_‐prepared bSSFP images overlayed on the corresponding images. The nnU‐Net model fails to segment the vessel at the last effective TE due to complete signal decay of the blood. In cases like these, the nnU‐Net segmentations were manually edited as needed.
**Figure S5:** Agreement in K‐MOTIVE metabolic measurements derived from manually and automatically segmented ROIs at four vascular sites in the testing datasets. A) T_2_, B) SvO_2_, C) BFR. The gray solid line is the line of identity. Each data point is the measurement from one K‐MOTIVE acquisition (PC images *n* = 180, T_2_‐prepared images *n* = 191).
**Table S1:** K‐MOTIVE pulse sequence parameters.
**Table S2:** Results from RM‐ANOVA, assessing within‐subject changes in metabolic parameters at each imaging location.
**Table S3:** nnU‐Net model performance evaluated using Dice score on testing data, for two models trained to segment K‐MOTIVE outputs at the left and right renal veins, as well as the suprarenal and infrarenal IVC.

## Data Availability

The source data is available to verified researchers upon request by contacting the corresponding author.

## References

[1] A. C. Hesp , J. A. Schaub , P. V. Prasad , et al., “The Role of Renal Hypoxia in the Pathogenesis of Diabetic Kidney Disease: A Promising Target for Newer Renoprotective Agents Including SGLT2 Inhibitors?,” Kidney International 98, no. 3 (2020): 579–589, 10.1016/j.kint.2020.02.041. 32739206 PMC8397597

[2] P. Hansell , W. J. Welch , R. C. Blantz , and F. Palm , “Determinants of Kidney Oxygen Consumption and Their Relationship to Tissue Oxygen Tension in Diabetes and Hypertension,” Clinical and Experimental Pharmacology & Physiology 40, no. 2 (2013): 123–137, 10.1111/1440-1681.12034. 23181475 PMC3951849

[3] A. Körner , A. C. Eklöf , G. Celsi , and A. Aperia , “Increased Renal Metabolism in Diabetes. Mechanism and Functional Implications,” Diabetes 43, no. 5 (1994): 629–633, 10.2337/diab.43.5.629. 8168637

[4] L. Nordquist , M. Friederich‐Persson , A. Fasching , et al., “Activation of Hypoxia‐Inducible Factors Prevents Diabetic Nephropathy,” Journal of the American Society of Nephrology 26, no. 2 (2015): 328–338, 10.1681/asn.2013090990. 25183809 PMC4310648

[5] M. Friederich‐Persson , P. Persson , P. Hansell , and F. Palm , “Deletion of Uncoupling Protein‐2 Reduces Renal Mitochondrial Leak Respiration, Intrarenal Hypoxia and Proteinuria in a Mouse Model of Type 1 Diabetes,” Acta Physiologica 223, no. 4 (2018): e13058, 10.1111/apha.13058. 29480974

[6] F. Palm , J. Cederberg , P. Hansell , P. Liss , and P. O. Carlsson , “Reactive Oxygen Species Cause Diabetes‐Induced Decrease in Renal Oxygen Tension,” Diabetologia 46, no. 8 (2003): 1153–1160, 10.1007/s00125-003-1155-z. 12879251

[7] A. Edwards and V. Kurtcuoglu , “Renal Blood Flow and Oxygenation,” Pflügers Archiv 474, no. 8 (2022): 759–770, 10.1007/s00424-022-02690-y. 35438336 PMC9338895

[8] L. G. Fine and J. T. Norman , “Chronic Hypoxia as a Mechanism of Progression of Chronic Kidney Diseases: From Hypothesis to Novel Therapeutics,” Kidney International 74, no. 7 (2008): 867–872, 10.1038/ki.2008.350. 18633339

[9] I. Mimura and M. Nangaku , “The Suffocating Kidney: Tubulointerstitial Hypoxia in End‐Stage Renal Disease,” Nature Reviews. Nephrology 6, no. 11 (2010): 667–678, 10.1038/nrneph.2010.124. 20877304

[10] B. Wang , Z. L. Li , Y. L. Zhang , Y. Wen , Y. M. Gao , and B. C. Liu , “Hypoxia and Chronic Kidney Disease,” EBioMedicine 77 (2022): 103942, 10.1016/j.ebiom.2022.103942. 35290825 PMC8921539

[11] T. Inoue , E. Kozawa , H. Okada , et al., “Noninvasive Evaluation of Kidney Hypoxia and Fibrosis Using Magnetic Resonance Imaging,” Journal of the American Society of Nephrology 22, no. 8 (2011): 1429–1434, 10.1681/asn.2010111143. 21757771 PMC3148697

[12] M. Pruijm , L. Hofmann , M. Piskunowicz , et al., “Determinants of Renal Tissue Oxygenation as Measured With BOLD‐MRI in Chronic Kidney Disease and Hypertension in Humans,” PLoS ONE 9, no. 4 (2014): e95895, 10.1371/journal.pone.0095895. 24760031 PMC3997480

[13] W. J. Yin , F. Liu , X. M. Li , et al., “Noninvasive Evaluation of Renal Oxygenation in Diabetic Nephropathy by BOLD‐MRI,” European Journal of Radiology 81, no. 7 (2012): 1426–1431, 10.1016/j.ejrad.2011.03.045. 21470811

[14] M. Pruijm , B. Milani , E. Pivin , et al., “Reduced Cortical Oxygenation Predicts a Progressive Decline of Renal Function in Patients With Chronic Kidney Disease,” Kidney International 93, no. 4 (2018): 932–940, 10.1016/j.kint.2017.10.020. 29325997

[15] C. Li , H. Liu , X. Li , L. Zhou , R. Wang , and Y. Zhang , “Application of BOLD‐MRI in the Classification of Renal Function in Chronic Kidney Disease,” Abdominal Radiology (NY) 44, no. 2 (2019): 604–611, 10.1007/s00261-018-1750-6. 30151714

[16] P. V. Prasad , “Update on Renal Blood Oxygenation Level‐Dependent MRI to Assess Intrarenal Oxygenation in Chronic Kidney Disease,” Kidney International 93, no. 4 (2018): 778–780, 10.1016/j.kint.2017.11.029. 29571450 PMC8438603

[17] T. Niendorf , A. Pohlmann , K. Arakelyan , et al., “How Bold Is Blood Oxygenation Level‐Dependent (BOLD) Magnetic Resonance Imaging of the Kidney? Opportunities, Challenges and Future Directions,” Acta Physiologica 213, no. 1 (2015): 19–38. 25204811 10.1111/apha.12393

[18] R. S. Deshpande , M. C. Langham , K. Susztak , and F. W. Wehrli , “MRI‐Based Quantification of Whole‐Organ Renal Metabolic Rate of Oxygen,” NMR in Biomedicine 37, no. 1 (2024): e5036, 10.1002/nbm.5036. 37750009 PMC10841084

[19] R. S. Deshpande , M. C. Langham , H. Lee , N. Kamona , and F. W. Wehrli , “Quantification of Whole‐Organ Individual and Bilateral Renal Metabolic Rate of Oxygen,” Magnetic Resonance in Medicine 91, no. 5 (2024): 2057–2073, 10.1002/mrm.29981. 38146669 PMC10950521

[20] N. Kamona , M. C. Langham , R. S. Deshpande , et al., “MRI‐Based Quantification of Whole‐Organ Renal Metabolic Rate of Oxygen During Free‐Breathing,” Magnetic Resonance in Medicine 94, no. 4 (2025): 1529–1545, 10.1002/mrm.30583. 40415411 PMC12309874

[21] R. G. Evans , D. Goddard , G. A. Eppel , and P. M. O'Connor , “Factors That Render the Kidney Susceptible to Tissue Hypoxia in Hypoxemia,” American Journal of Physiology. Regulatory, Integrative and Comparative Physiology 300, no. 4 (2011): R931–R940, 10.1152/ajpregu.00552.2010. 21248306

[22] R. G. Evans , C. Ince , J. A. Joles , et al., “Haemodynamic Influences on Kidney Oxygenation: Clinical Implications of Integrative Physiology,” Clinical and Experimental Pharmacology & Physiology 40, no. 2 (2013): 106–122, 10.1111/1440-1681.12031. 23167537

[23] D. H. Wong , P. D. Weir , R. C. Wesley , et al., “Changes in Renal Vein, Renal Surface, and Urine Oxygen Tension During Hypoxia in Pigs,” Journal of Clinical Monitoring 9, no. 1 (1993): 1–4, 10.1007/bf01627629. 8463800

[24] J. B. West , Pulmonary Physiology and Pathophysiology: An Integrated, Case‐Based Approach (Wolters Kluwer Health/Lippincott Williams & Wilkins, 2007).

[25] T. H. Allen , H. J. Krzywicki , and J. E. Roberts , “Density, Fat, Water and Solids in Freshly Isolated Tissues,” Journal of Applied Physiology 14 (1959): 1005–1008, 10.1152/jappl.1959.14.6.1005. 13792786

[26] W. Li and P. C. M. van Zijl , “Quantitative Theory for the Transverse Relaxation Time of Blood Water,” NMR in Biomedicine 33, no. 5 (2020): e4207, 10.1002/nbm.4207. 32022362 PMC7322972

[27] W. Li , K. Grgac , A. Huang , N. Yadav , Q. Qin , and P. C. van Zijl , “Quantitative Theory for the Longitudinal Relaxation Time of Blood Water,” Magnetic Resonance in Medicine 76, no. 1 (2016): 270–281, 10.1002/mrm.25875. 26285144 PMC4758918

[28] D. Y. Balaban , J. Duffin , D. Preiss , et al., “The In‐Vivo Oxyhaemoglobin Dissociation Curve at Sea Level and High Altitude,” Respir Physiol Neurobiol 186, no. 1 (2013): 45–52, 10.1016/j.resp.2012.12.011. 23313855

[29] J. W. Severinghaus , “Simple, Accurate Equations for Human Blood O2 Dissociation Computations,” Journal of Applied Physiology: Respiratory, Environmental and Exercise Physiology 46, no. 3 (1979): 599–602, 10.1152/jappl.1979.46.3.599. 35496

[30] J. A. Fessler and B. P. Sutton , “Nonuniform Fast Fourier Transforms Using Min‐Max Interpolation,” IEEE Transactions on Signal Processing 51, no. 2 (2003): 560–574, 10.1109/TSP.2002.807005.

[31] K. P. Pruessmann , M. Weiger , M. B. Scheidegger , and P. Boesiger , “SENSE: Sensitivity Encoding for Fast MRI,” Magnetic Resonance in Medicine 42, no. 5 (1999): 952–962. 10542355

[32] K. P. Pruessmann , M. Weiger , P. Börnert , and P. Boesiger , “Advances in Sensitivity Encoding With Arbitrary K‐Space Trajectories,” Magnetic Resonance in Medicine 46, no. 4 (2001): 638–651, 10.1002/mrm.1241. 11590639

[33] F. Ong and M. Lustig , SigPy: A Python Package for High Performance Iterative Reconstruction. Presented at: ISMRM Annual Meeting (International Society of Magnetic Resonance in Medicine: 2019).

[34] F. Isensee , P. F. Jaeger , S. A. A. Kohl , J. Petersen , and K. H. Maier‐Hein , “NnU‐Net: A Self‐Configuring Method for Deep Learning‐Based Biomedical Image Segmentation,” Nature Methods 18, no. 2 (2021): 203–211, 10.1038/s41592-020-01008-z. 33288961

[35] I. N. De Keijzer , D. Massari , C. K. Niezen , R. P. H. Bokkers , J. J. Vos , and T. W. L. Scheeren , “Agreement of Somatic and Renal Near‐Infrared Spectroscopy With Reference Blood Samples During a Controlled Hypoxia Sequence: A Healthy Volunteer Study,” Journal of Clinical Monitoring and Computing 37, no. 3 (2023): 805–814, 10.1007/s10877-022-00944-9. 36463540 PMC10175462

[36] B. R. Kurnik , L. S. Weisberg , and P. B. Kurnik , “Renal and Systemic Oxygen Consumption in Patients With Normal and Abnormal Renal Function,” Journal of the American Society of Nephrology : JASN 2, no. 11 (1992): 1617–1626, 10.1681/asn.V2111617. 1610983

[37] Z. Ren , S. L. Wang , and M. A. Singer , “Modeling Hemodynamics in an Unoccluded and Partially Occluded Inferior Vena Cava Under Rest and Exercise Conditions,” Medical & Biological Engineering & Computing 50, no. 3 (2012): 277–287, 10.1007/s11517-012-0867-y. 22354383

[38] E. F. Cox , C. E. Buchanan , C. R. Bradley , et al., “Multiparametric Renal Magnetic Resonance Imaging: Validation, Interventions, and Alterations in Chronic Kidney Disease,” Frontiers in Physiology 8 (2017): 696, 10.3389/fphys.2017.00696. 28959212 PMC5603702

[39] C. P. Cheng , R. J. Herfkens , and C. A. Taylor , “Inferior Vena Caval Hemodynamics Quantified In Vivo at Rest and During Cycling Exercise Using Magnetic Resonance Imaging,” American Journal of Physiology. Heart and Circulatory Physiology 284, no. 4 (2003): H1161–H1167, 10.1152/ajpheart.00641.2002. 12595296

[40] A. A. Joseph , D. Voit , and J. Frahm , “Inferior Vena Cava Revisited ‐ Real‐Time Flow MRI of Respiratory Maneuvers,” NMR in Biomedicine 33, no. 4 (2020): e4232, 10.1002/nbm.4232. 31913551

[41] Y. Jung , H. S. Ahn , and S. H. Park , “Quantitative Mapping of Renal Oxygen Consumption Using Pseudo‐Continuous Arterial Spin Labeling and Quantitative Susceptibility Mapping in Humans,” Magnetic Resonance in Medicine 93, no. 2 (2025): 699–708, 10.1002/mrm.30288. 39221556

[42] S.‐E. Ricksten , G. Bragadottir , L. Lannemyr , B. Redfors , and J. Skytte , “Renal Hemodynamics, Function, and Oxygenation in Critically Ill Patients and After Major Surgery,” Kidney360 2, no. 5 (2021): 894–904, 10.34067/kid.0007012020. 35373068 PMC8791344

[43] R. W. Gotshall , D. S. Miles , and W. R. Sexson , “Renal Oxygen Delivery and Consumption During Progressive Hypoxemia in the Anesthetized Dog,” Proceedings of the Society for Experimental Biology and Medicine 174, no. 3 (1983): 363–367, 10.3181/00379727-174-41749. 6420794

[44] J. Carr , R. L. Hoiland , I. A. Fernandes , W. G. Schrage , and P. N. Ainslie , “Recent Insights Into Mechanisms of Hypoxia‐Induced Vasodilatation in the Human Brain,” Journal of Physiology 602, no. 21 (2024): 5601–5618, 10.1113/jp284608. 37655827

[45] F. A. Dinenno , “Skeletal Muscle Vasodilation During Systemic Hypoxia in Humans,” Journal of Applied Physiology (1985) 120, no. 2 (2016): 216–225, 10.1152/japplphysiol.00256.2015. PMC4719058 26023228

[46] R. G. Evans , D. W. Smith , C. J. Lee , J. P. Ngo , and B. S. Gardiner , “What Makes the Kidney Susceptible to Hypoxia?,” Anatomical Record (Hoboken) 303, no. 10 (2020): 2544–2552, 10.1002/ar.24260. 31566903

[47] A. Pohlmann , K. Arakelyan , J. Hentschel , et al., “Detailing the Relation Between Renal T2* and Renal Tissue pO2 Using an Integrated Approach of Parametric Magnetic Resonance Imaging and Invasive Physiological Measurements,” Investigative Radiology 49, no. 8 (2014): 547–560, 10.1097/rli.0000000000000054. 24651661

[48] D. Grosenick , K. Cantow , K. Arakelyan , et al., “Detailing Renal Hemodynamics and Oxygenation in Rats by a Combined Near‐Infrared Spectroscopy and Invasive Probe Approach,” Biomedical Optics Express 6, no. 2 (2015): 309–323, 10.1364/boe.6.000309. 25780726 PMC4354597

[49] R. A. Sharkey , E. M. Mulloy , and S. J. O'Neill , “Acute Effects of Hypoxaemia, Hyperoxaemia and Hypercapnia on Renal Blood Flow in Normal and Renal Transplant Subjects,” European Respiratory Journal 12, no. 3 (1998): 653–657, 10.1183/09031936.98.12030653. 9762795

[50] Q. Xu , D. Jiang , Y.‐C. Hsu , et al., “Noninvasive Quantification of Renal Venous Oxygenation With Field‐Insensitive T2 Preparation and Fast Acquisition,” Magnetic Resonance in Medicine 94 (2025): 2447–2459, 10.1002/mrm.70010. 40712093

[51] P. V. Prasad , L. P. Li , B. Hack , N. Leloudas , and S. M. Sprague , “Quantitative Blood Oxygenation Level Dependent Magnetic Resonance Imaging for Estimating Intra‐Renal Oxygen Availability Demonstrates Kidneys Are Hypoxemic in Human CKD,” Kidney International Reports 8, no. 5 (2023): 1057–1067, 10.1016/j.ekir.2023.02.1092. 37180507 PMC10166744

