# Supplementary material for: Renal Metabolic Rate of Oxygen in Response to Hypoxia Challenges by Means of Quantitative MRI in Humans
Source: NMR Biomed. 2025 Nov 14;38(12):e70178. doi: 10.1002/nbm.70178 (PMC12616586; doi:10.1002/nbm.70178)
Supplement: Supplementary file 1 — Figure S1: Overview of the K‐MOTIVE MRI method (Kidney Metabolism of Oxygen via T2 and Interleaved Velocity Encoding), designed to noninvasively estimate renal metabolic rate of oxygen (rMRO2) in vivo. Figure S2: Example segmentation of five K‐MOTIVE PC images overlayed on the corresponding magnitude images, demonstrating high agreement with the ground‐truth manual segmentation. Figure S3: Example segmentation of five K‐MOTIVE T2‐prepared bSSFP images overlayed on the corresponding images, demonstrating high agreement with the ground‐truth manual segmentation. Figure S4: Example segmentation of five K‐MOTIVE T2‐prepared bSSFP images overlayed on the corresponding images. The nnU‐Net model fails to segment the vessel at the last effective TE due to complete signal decay of the blood. In cases like these, the nnU‐Net segmentations were manually edited as needed. Figure S5: Agreement in K‐MOTIVE metabolic measurements derived from manually and automatically segmented ROIs at four vascular sites in the testing datasets. A) T2, B) SvO2, C) BFR. The gray solid line is the line of identity. Each data point is the measurement from one K‐MOTIVE acquisition (PC images n = 180, T2‐prepared images n = 191). Table S1: K‐MOTIVE pulse sequence parameters. Table S2: Results from RM‐ANOVA, assessing within‐subject changes in metabolic parameters at each imaging location. Table S3: nnU‐Net model performance evaluated using Dice score on testing data, for two models trained to segment K‐MOTIVE outputs at the left and right renal veins, as well as the suprarenal and infrarenal IVC. [file NBM-38-e70178-s001.docx]

**Supplementary Material**

**Title:**

Renal metabolic rate of oxygen in response to hypoxia challenges by means of quantitative MRI in humans

**Authors:**

Nada Kamona, MS^1,2^, Mahdie Hosseini, MD^1^, Michael C. Langham, PhD^1^, Felix W. Wehrli, PhD^1^

*^1^Department of Radiology, Perelman School of Medicine, University of Pennsylvania, Philadelphia, Pennsylvania, USA*

*^2^Department of Bioengineering, School of Engineering and Applied Sciences, University of Pennsylvania, Philadelphia, Pennsylvania, USA*

**Table of Content**

| **Topic** | **Page Number** |
| --- | --- |
| K-MOTIVE Method Overview & Sequence Parameters | 2 |
| RM-ANOVA Results | 4 |
| Deep-learning Model for Automatic Vessel Segmentation | 5 |
| Additional References | 7 |

**K-MOTIVE Method Overview & Sequence Parameters**


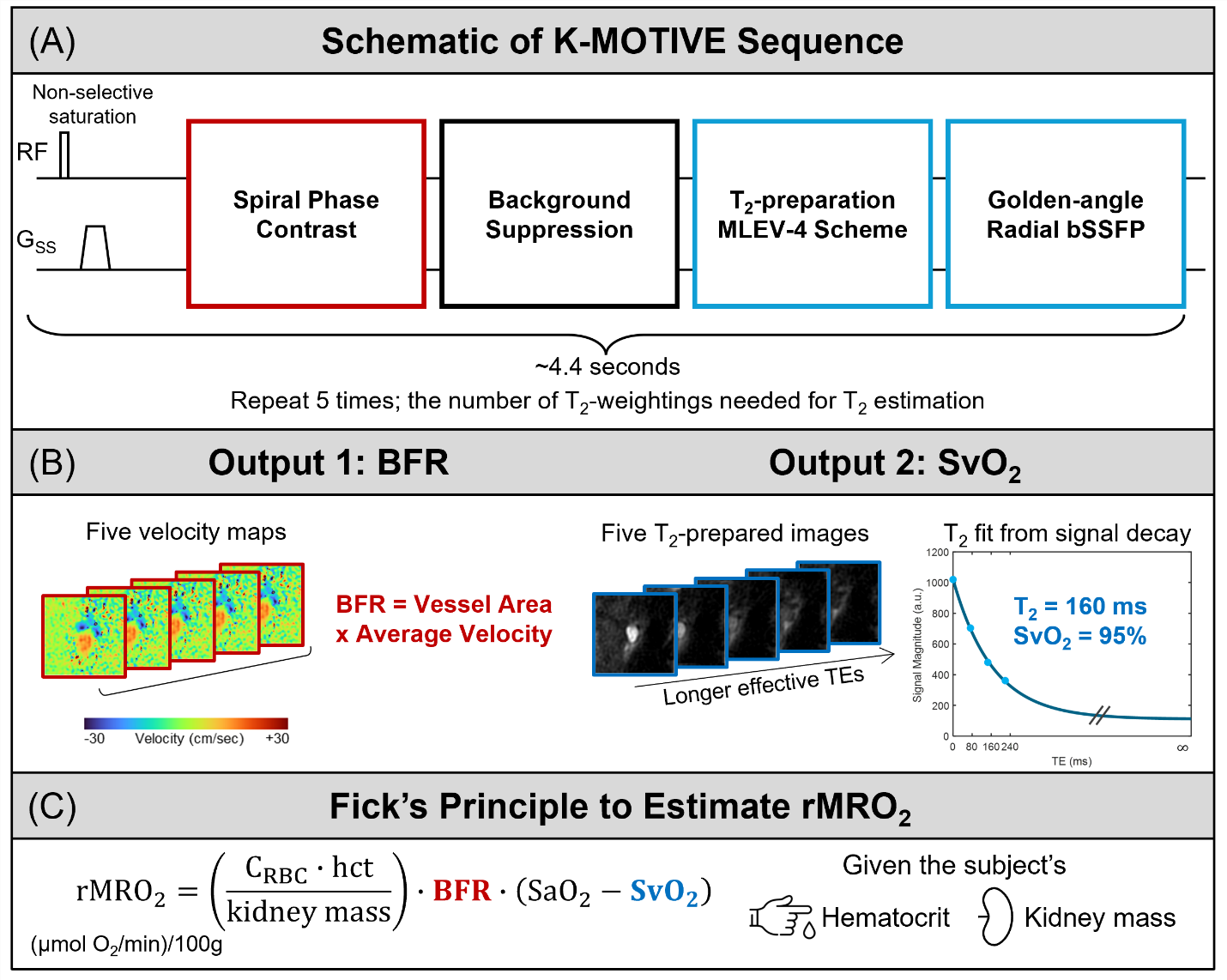


**Figure S1:** Overview of the K-MOTIVE MRI method (Kidney Metabolism of Oxygen via T_2_ and Interleaved Velocity Encoding), designed to noninvasively estimate renal metabolic rate of oxygen (rMRO_2_) in vivo. **A)** Simplified schematic of the pulse sequence^1^. Each pass of the sequence is repeated five times in total to encode longer T_2_-weighting in the longitudinal magnetization. **B)** K-MOTIVE outputs five velocity maps and five T_2_-prepared images, from which blood flow rate (BFR) and T_2_ of blood water are calculated. T_2_ is then converted to venous oxygen saturation (SvO_2_) using a calibration curve^2^. **C)** rMRO_2_ is estimated from conservation of mass, or Fick’s Principle, using the BFR and SvO_2_ outputs from K-MOTIVE. Subject-specific hematocrit is obtained via a finger prick, kidney mass is estimated from a separate kidney anatomical scan and known tissue density, and arterial oxygen saturation (SaO_2_) is measured via a pulse oximeter.

**Table S1:** K-MOTIVE pulse sequence parameters, previously reported in Kamona et al. ^1^.

| **Sequence Module** | **Module Parameters** |
| --- | --- |
| Phase contrast spiral readout | Constant linear speed spiral; Number spiral shots per interleaf = 51; Number of readout samples = 1530; Flip angle = 15°; TR/TE = 12.5/4 ms; VENC = 50 cm/s ; Slice thickness = 5 mm; FOV = 304 × 304 mm^2^; Matrix size = 608 × 608, grid size factor = 2; Voxel size = 1 × 1 × 5 mm^3^; Max gradient amplitude = 19.4 mT/m, max slew rate = 0.095 [mT/m]/µs; kmax = 500 m^-1^, bandwidth/pix = 164 Hz; Total acquisition time = 2.15 s (70 dummy TRs at the beginning with total time of 0.88 s, followed by 102 TRs are used for image reconstruction with total acquisition time of 1.28s); T_sat_ = 2500 ms |
| Background suppression | Slice-selective saturation followed by 4 adiabatic inversion pulses: 90°_SS_ - 180°_SS_ - 180°_SS_ - 180°_NS_ - 180°_NS_; where SS is slice-selective, NS is non-selective; Ratio of inversion slab thickness to T2-prepared bSSFP imaging slice thickness=1.5; SS inversion slab thickness=18mm; Total duration = 1000 ms; Pulse times 0, 140, 450, 750, 940 ms |
| T_2_-preparation | Composite pulses: 90°_x_…(90°_x_180°_y_90°_x_)^4n^…270°_x_360°_-x_ ; MLEV-4 pattern: +y, +y, -y, -y;  All non-selective rectangular pulses; Five interleaves: n = 0, 1, 2, 3, sat. The saturated magnetization is for a three-parameter fit of bSSFP signal; Composite pulse duration τ_p_ = 2.3 ms; Inter-pulse interval τ_180_ = 12 or 20 ms; T_2_-preparation times for τ_180_ of 12 ms = 0, 48, 96, 144, and ∞ ms; T_2_-preparation times for τ_180_ of 20 ms = 0, 80, 160, 240, and ∞ ms |
| bSSFP golden-angle radial readout | Flip angle = 60°; TR/TE = 4.50/2.25 ms; FOV = 300 × 300 mm^2^; Slice thickness = 12 mm; Voxel size = 1.5 × 1.5 × 12 mm^2^; Number of radial lines = 144, each crossing the center of k-space; Golden-angle = 111.25°; 10 catalyzation TRs; Total duration (acquisition window) = 0.6 seconds |
| Abbreviations: bSSFP, balanced steady-state free precession; FOV, field of View; MLEV-4, Malcolm-Levitt T2-preparation scheme; VENC, velocity-encoding parameter; T_sat_, time between global saturation and start of the background suppression module. | |

**Table S2:** Results from RM-ANOVA, assessing within-subject changes in metabolic parameters at each imaging location.

| **Imaging location** | **Parameter** ^α^ | **F(DFn, DFd)** ^γ^ | **p-value** | **Generalized η^2^ measure of effect size** |
| --- | --- | --- | --- | --- |
| Left | T_2_ | F(3,24) = 71.4 | **<0.001** | 0.81 |
|  | SvO_2_ | F(3,24) = 72.3 | **<0.001** | 0.75 |
|  | AVDO_2_ | F(3,24) = 0.13 | 0.94 | 0.01 |
|  | BFR | F(3,24) = 4.63 | **0.011** | 0.05 |
|  | rMRO_2_ | F(3,24) = 1.02 | 0.40 | 0.05 |
| Suprarenal IVC | T_2_ | F(3,24) = 18.2 | **<0.001** | 0.66 |
|  | SvO_2_ | F(3,24) = 24.8 | **<0.001** | 0.69 |
|  | AVDO_2_ | F(3,24) = 1.49 | 0.24 | 0.11 |
|  | BFR | F(1.8, 14.4) = 6.56 ^δ^ | **0.011** | 0.15 |
| Infrarenal IVC | T_2_ | F(3,24) = 9.73 | **<0.001** | 0.30 |
|  | SvO_2_ | F(3,24) = 5.64 | **0.005** | 0.28 |
|  | AVDO_2_ | F(3,24) = 1.12 | 0.36 | 0.07 |
|  | BFR | F(1.6, 12.7) = 3.50 ^δ^ | 0.07 | 0.11 |
| Bilateral (Suprarenal – Infrarenal IVC) | ∆ BFR ^β^ | F(3,24) = 2.30 | 0.10 | 0.10 |
|  | rMRO_2_ | F(1.1, 9.1) = 0.85 ^δ^ | 0.40 | 0.07 |
| ^α^ RM-ANOVA was constructed for each metabolic parameter and imaging location for nine subjects. Due to scanner technical difficulties, one subject was not scanned during the recovery stage. Consequently, data from this participant was included in descriptive summaries but excluded from RM-ANOVA analyses.  ^β^ The bilateral BFR is defined as the BFR difference between suprarenal and infrarenal IVC.  ^γ^ The degrees of freedom in numerator were calculated as (k-1) and the degrees of freedom in denominator were calculated as (n-1)(k-1), where n=number of subjects and k=number of conditions. In this case, n=9 and k=4.  ^δ^ Mauchly’s sphericity test was violated with p<0.05. Greenhouse-Geisser was used to adjust the degrees of freedom and p-values for the F-test.  Abbreviations: DFn, degrees of freedom in numerator; DFd, degrees of freedom in denominator; SaO_2_, arterial oxygen saturation; SvO_2_, venous oxygen saturation; AVDO_2_, arteriovenous difference in oxygen saturation (SaO_2_ – SvO_2_); BFR, blood flow rate; rMRO_2_, renal metabolic rate of oxygen. | | | | |

**Deep-learning Model for Automatic Vessel Segmentation**

An automated segmentation pipeline based on the nnU-Net ("no-new" U-Net) framework^3^ was developed to segment renal veins and the IVC from K-MOTIVE images. The nnU-Net, based on the U-Net architecture, is composed of encoder, decoder, and skip connections. It has the advantage of combining both low- and high-resolution information, and thus, has good generalization even without large training datasets. Most importantly, the nnU-Net is a self-configured deep-learning method with an automated end-to-end pipeline, which avoids the need of manually fine-tuning the model parameters as is mostly done in deep-learning segmentation tasks.

Training data were collected from 15 healthy adults, previously reported in Kamona et al.^1^. K-MOTIVE was acquired 18-24 times per subject (22-seconds per acquisition) at four imaging sites: left and right veins, and suprarenal and infrarenal IVC. Only seven out of 15 subjects were scanned at the right renal vein due to the vessels being much shorter than the corresponding left side (see Kamona et al. for details^1^). Half of the scans were performed during free breathing, and the other half during breath-holding. Each K-MOTIVE acquisition generated five T_2_-prepared bSSFP images and five PC velocity maps. Manual segmentations were used as ground-truth.

The nnU-Net models were implemented as provided in Isensee et al.^3^, using the first available version on the authors provided GitHub codes.

Two separate 2D nnU-Net models were trained to segment the two K-MOTIVE outputs:

1. The first model was trained to segment 2D PC images across four vascular sites. Training data included 329 PC datasets from 10 subjects, each comprising five slices with two channels (magnitude and velocity), resulting in an input size of 1216 × 1216 × 5 × 2. Testing was performed on 180 datasets from five independent subjects not seen during training.
2. The second model was trained to segment 2D T_2_-prepared bSSFP images across four vascular sites. Training used 332 datasets from 10 subjects, with each dataset comprising five single-channel slices of input size: 400 × 400 × 5. Testing was performed on 191 datasets from five additional subjects not seen during training.

The hypoxia experiment data collected in the presented manuscript was not included in the training/testing datasets for the nnU-Net models.

**Table S3:** nnU-Net model performance evaluated using Dice score on testing data, for two models trained to segment K-MOTIVE outputs at the left and right renal veins, as well as the suprarenal and infrarenal IVC.

| **Dice Score** | **PC Images** | | | **T_2_-prepared bSSFP images** | | |
| --- | --- | --- | --- | --- | --- | --- |
|  | **Mean ± STD** | **Median** | ***n*** | **Mean ± STD** | **Median** | ***n*** |
| Overall for all vessels | 0.89 ± 0.11 | 0.92 | 180 | 0.83 ± 0.11 | 0.86 | 191 |
| Left renal vein | 0.89 ± 0.11 | 0.92 | 45 | 0.78± 0.13 | 0.83 | 50 |
| Right renal vein | 0.78 ± 0.14 | 0.80 | 36 | 0.80± 0.08 | 0.80 | 36 |
| Suprarenal IVC | 0.94 ± 0.04 | 0.95 | 51 | 0.91± 0.12 | 0.85 | 51 |
| Infrarenal IVC | 0.92 ± 0.06 | 0.95 | 48 | 0.84± 0.06 | 0.95 | 54 |

**
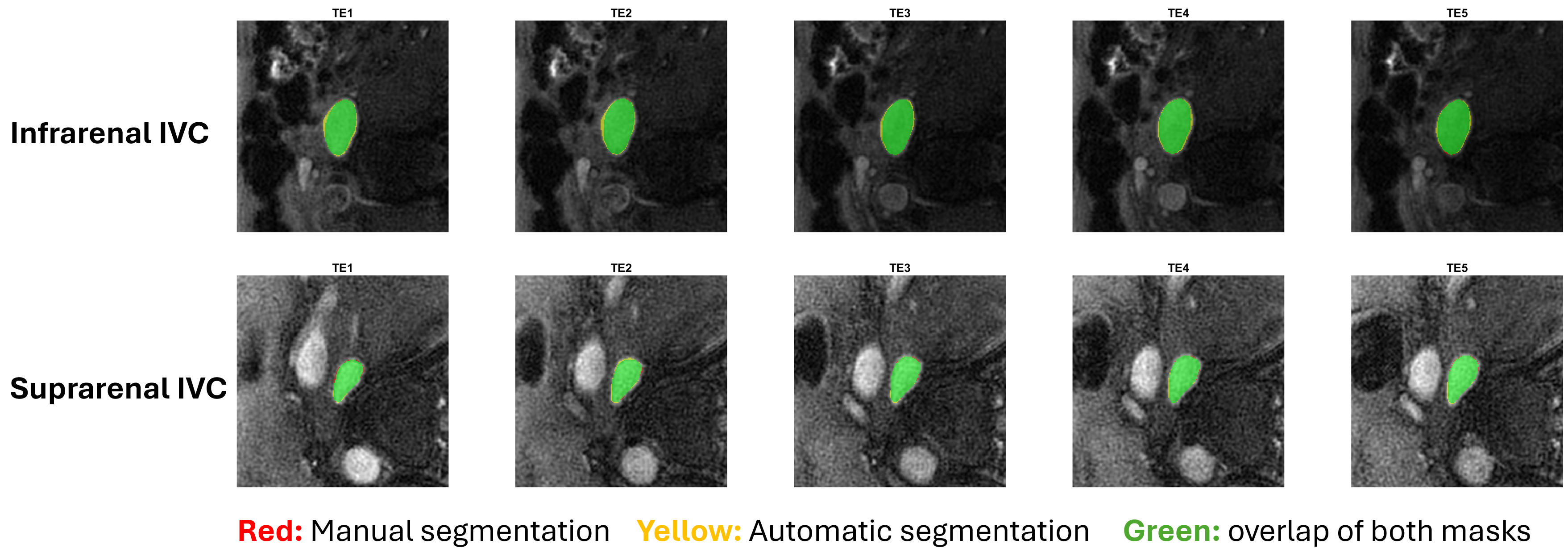
**

**Figure S2:** Example segmentation of five K-MOTIVE PC images overlayed on the corresponding magnitude images, demonstrating high agreement with the ground-truth manual segmentation.

**
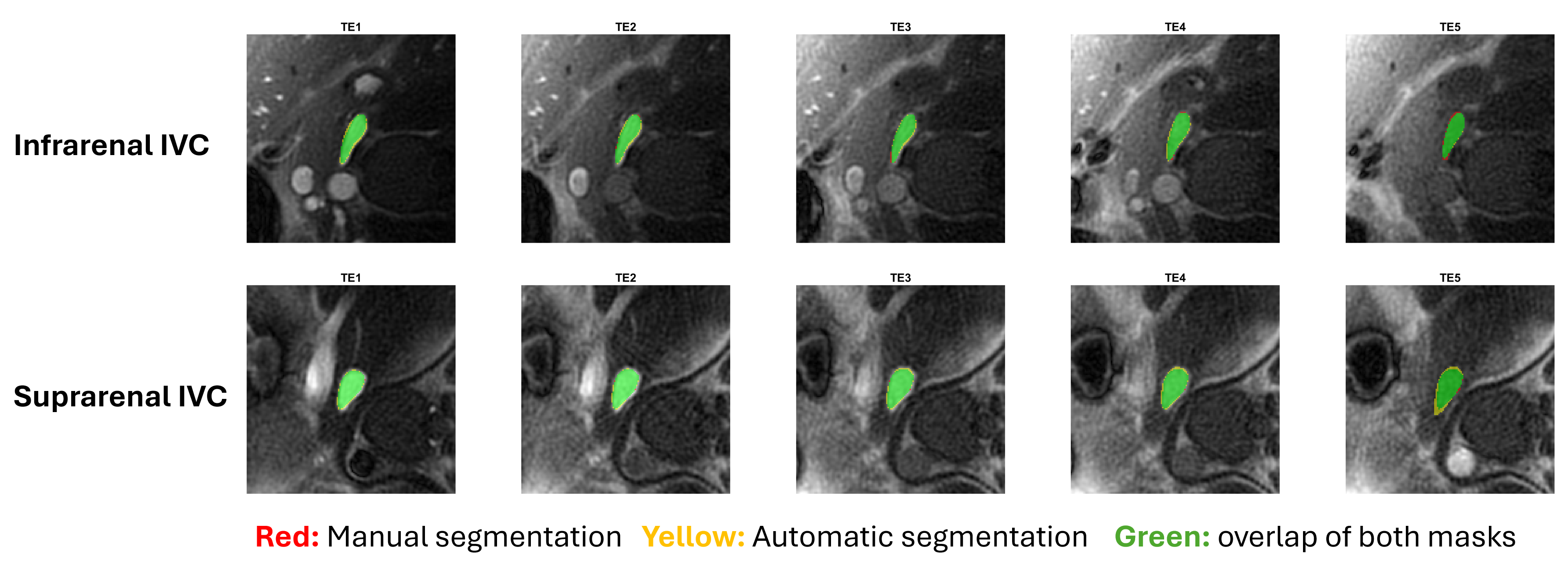
**

**Figure S3:** Example segmentation of five K-MOTIVE T_2_-prepared bSSFP images overlayed on the corresponding images, demonstrating high agreement with the ground-truth manual segmentation.


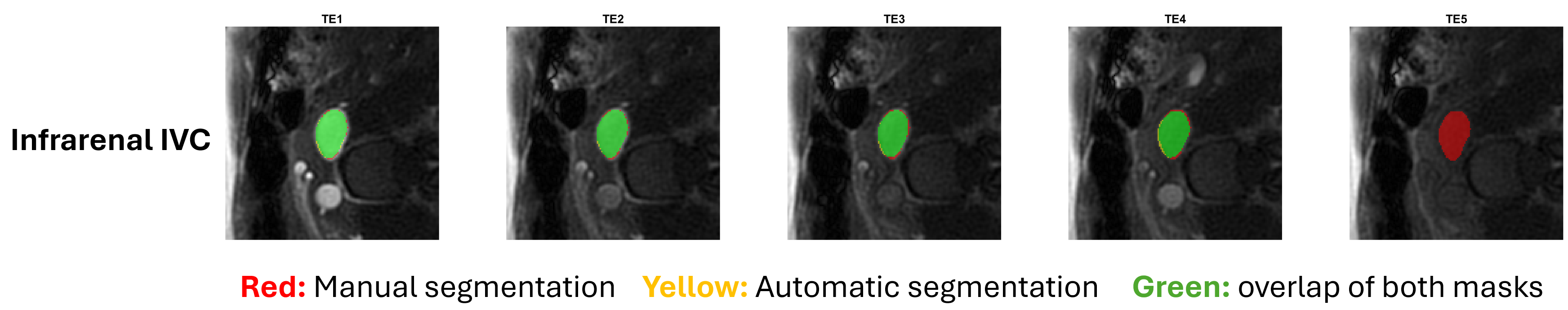


**Figure S4:** Example segmentation of five K-MOTIVE T_2_-prepared bSSFP images overlayed on the corresponding images. The nnU-Net model fails to segment the vessel at the last effective TE due to signal decay of the blood. *In cases like these, the nnU-Net segmentations were manually edited as needed.*

To quantitatively evaluate the nnU-Net model outputs, metabolic parameters were derived from the automatic segmentations and were compared to those obtained from the manual ROIs. Overall good concordance was observed across all metabolic parameters between the two segmentations across all vascular sites, as measured by Lin’s concordance correlation coefficient (CCC). Specifically, at the IVC, there was good agreement for T_2_ (CCC>0.90), SvO_2_ (CCC>0.91), and BFR (CCC>0.98). The renal veins had overall good agreement across all metabolic parameters (CCC>0.75), except for BFR at the right renal vein which had poor agreement (CCC=0.40) between manual and automatic segmentations due to the small size available at the right renal vein. *The automatic segmentations in this agreement analysis were not manually edited.*


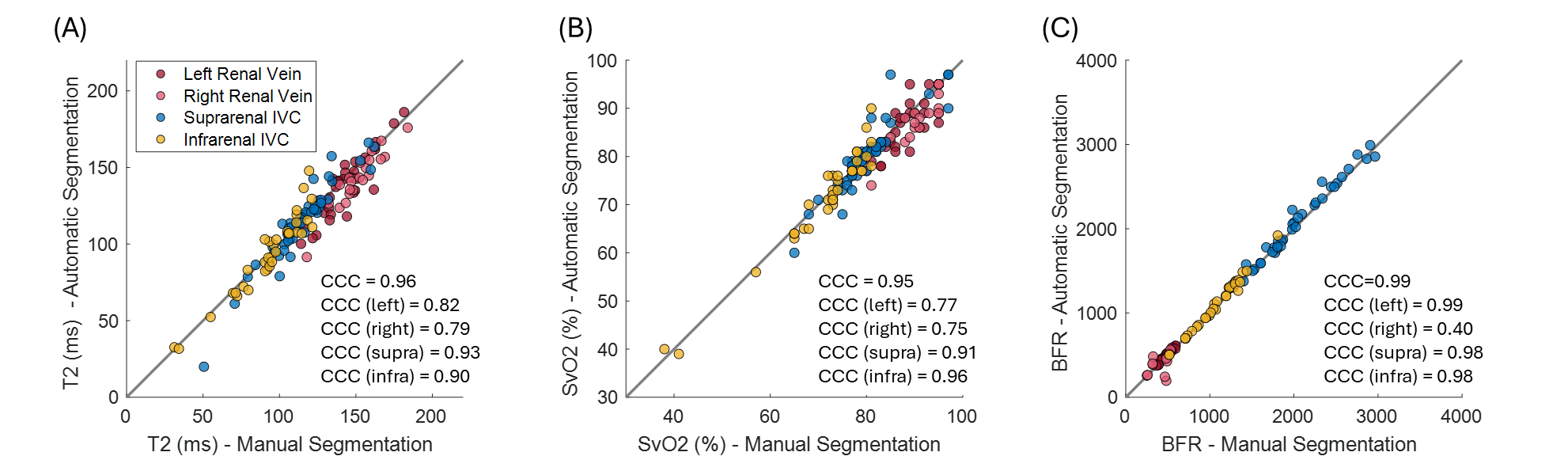


**Figure S5**: Agreement in K-MOTIVE metabolic measurements derived from manually and automatically segmented ROIs at four vascular sites in the testing datasets. **A)** T_2_, **B)** SvO_2_, **C)** BFR. The gray solid line is the line of identity. Each data point is the measurement from one K-MOTIVE acquisition (PC images n=180, T_2_-prepared images n =191).

**Supplementary References:**

1. Kamona N, Langham MC, Deshpande RS, Vu B-TD, Hosseini M, Dennison J, Jaroszynski KM, Wehrli FW. MRI-based quantification of whole-organ renal metabolic rate of oxygen during free-breathing. Magnetic Resonance in Medicine. 2025. doi: <https://doi.org/10.1002/mrm.30583>.

2. Li W, van Zijl PCM. Quantitative theory for the transverse relaxation time of blood water. NMR Biomed. 2020;33(5):e4207. Epub 20200205. doi: 10.1002/nbm.4207. PubMed PMID: 32022362; PMCID: PMC7322972.

3. Isensee F, Jaeger PF, Kohl SAA, Petersen J, Maier-Hein KH. nnU-Net: a self-configuring method for deep learning-based biomedical image segmentation. Nature Methods. 2021;18(2):203-11. doi: 10.1038/s41592-020-01008-z.
